# Chronic Proliferative Dermatitis in Mice: NF**κ**B Activation Autoinflammatory Disease

**DOI:** 10.4061/2011/936794

**Published:** 2011-06-01

**Authors:** Yanhua Liang

**Affiliations:** ^1^Department of Dermatology, Yale University School of Medicine, 15 York Street, New Haven, CT 06510, USA; ^2^The Jackson Laboratory, Bar Harbor, ME 04609, USA

## Abstract

Autoinflammatory diseases are a heterogeneous group of congenital diseases characterized by the presence of recurrent inflammation, in the absence of infectious agents, detectable autoantibodies or antigen-specific autoreactive T-cells. SHARPIN deficient mice presents multiorgan chronic inflammation without known autoantibodies or autoreactive T-cells, designated *Sharpin^cpdm^*. Histological studies demonstrated epidermal hyperproliferation, Th-2 inflammation, and keratinocyte apoptosis in this mutant. The mutant mice have decreased behavioral mobility, slower growth, and loss of body weight. Epidermal thickness and mitotic epidermal cells increase along with disease development. K5/K14 expression is distributed through all layers of epidermis, along with K6 expression in interfollicular epidermis, suggesting epidermal hyperproliferation. K1/K10 is only detectable in outer layers of spinosum epidermis, reflecting accelerated keratinocyte migration. Alpha smooth muscle actin is overexpressed in skin blood vessels, which may release the elevated white blood cells to dermis. CD3^+^CD45^+^ cells and granulocytes, especially eosinophils and mast cells, aggregate in the mutant skin. TUNEL assay, together with Annexin-V/propidium iodide FACS analysis, confirmed the increase of apoptotic keratinocytes in skin. These data validate and provide new lines of evidence of the proliferation-inflammation-apoptosis triad in *Sharpin^cpdm^* mice, an NF*κ*B activation autoinflammatory disease.

## 1. Introduction

The relatively new term of “autoinflammatory disease” appeared in the pages of “Cell” in the spring of 1999 to denote an emerging group of heritable disorders characterized by two negatives, the lack of apparent provocation of seemingly unprovoked inflammation, and the absence of high-titer autoantibodies or antigen-specific T cells [1, 2]. Although different from autoimmune diseases, autoinflammatory and autoimmune diseases share common characteristics in that both groups of disorders result from the immune system attacking the body's own tissues and also result in increased inflammation. Simply put, the autoinflammatory diseases are clinical disorders marked by abnormally increased inflammation, mediated predominantly by the cells and molecules of the innate immune system, with a significant host predisposition [3]. The molecular pathophysiology of the autoinflammatory diseases has been a topic of growing clinical interest. Positional cloning and candidate gene analysis have provided important insights to the monogenic autoinflammatory diseases. In the last decade, common variants by genome-wide association studies have been detected as the genetic basis of complex autoinflammatory diseases. The recently discovered genetic bases of autoinflammatory diseases have been linked to the mutations in genes encoding a relatively new family of proteins that have important roles in the regulation of apoptosis, inflammation, and cytokine processing [4]. A good example in this context is the association of *CARD15* and *NOD2* variations with the Blau syndrome and Crohn's disease [5, 6].

Chronic proliferative dermatitis mutant (*cpdm*) mice are characterized by multiorgan inflammation initially recognized as erythematous, scaly areas of alopecia at 3-4 weeks of age [7]. Both clinical features and histological changes, such as epidermal hyperplasia and severe dermal inflammation, made investigators have taken *cpdm *mice as a model of human psoriasis, hence alternatively named “psoriasiform dermatitis” [8, 9]. Even considering the anatomic and biological differences between human and mouse [10], normal keratinocyte differentiation [11], Th2 but not Th1 inflammation [12], and different malfunctions of involved organs suggested *Sharpin^cpdm^* mice do not indeed resemble human psoriasis. Beside dermatitis, hepatosplenomegaly, pneumonia, lymphadenitis, upper gastrointestinal eosinophilic inflammation, and osteopenic phenotype are also severe symptoms in *Sharpin^cpdm^* mice [13–15]. Persistent eosinophilia, multisystem eosinophil infiltration, aberrant CD3^−^CD4^+^ cells, equivalent sex ratio, and Th2-type inflammation suggested *Sharpin^cpdm^* mutant may be more likely as a mouse model for the lymphocytic variant of hypereosinophilic syndrome (LHES) [14].

Positional cloning studies identified two independent, spontaneous autosomal recessive mutations in the mice that were null for the *Sharpin* gene product as the genomic defect of this phenotype [13]. SHARPIN is widely expressed in multiple tissues in human and mice [16]. Protein motif search suggested three important functional domains of SHARPIN, the Hoil1-N domain of ubiquitination activity, region AA172-305 interacting with SHANK1, and C terminal RanBP domain associated with nuclear transport. A yeast two-hybrid (Y2H) screen with TRAF2 (TNF receptor-associated factor 2) as the bait (UCSD Nature Signaling Gateway, data center, yeast two-hybrid, http://www.signaling-gateway.org/data/Y2H/cgi-bin/y2h _int.cgi?id=53738) has identified SHARPIN (alternative symbol: protein kinase C-interacting protein RBCC like 1 (RBCKL1)) is one of its preys in B cell lines. Protein immunoprecipitation identified SHARPIN has interaction with TRAF2 and negatively regulates TRAF2-mediated NF*κ*B activity [17]. Inhibition of NF*κ*B signaling can significantly alleviate the skin problems in *Sharpin^cpdm^* mice [14]. 

Many physiological processes, including proper tissue development and homeostasis, require a balance between apoptosis and cell proliferation. All somatic cells proliferate via a mitotic process determined by progression through the cell cycle. Apoptosis and program cell death (PCD) is a genetically regulated, cellular suicide mechanism which occurs in a wide variety of physiological settings, where its role is to remove harmful, damaged, or unwanted cells. Apoptosis and cell proliferation are linked by cell-cycle regulators and apoptotic stimuli that affect both processes [18]. Imbalance of proliferation and apoptosis is commonly seen in various skin diseases such as psoriasis and sunburn [19]. In mammalian cells, a variety of apoptotic stimuli induce a series of biochemical reactions that imbalance the ratio of proapoptotic/antiapoptotic (e.g., Bax/Bcl2 ratio) proteins between the mitochondria and cytosol and then disturb energy metabolism resulting mitochondria defect by releasing death factors into the cytosol and then leading to the formation of apoptosome and activation of caspases resulting in apoptosis. The spontaneous null mutation of *Sharpin *(*Sharpin^cpdm^*) causes mice to undergo abnormal epithelial cell death in the skin, esophagus, and forestomach [20]. As the balance of epidermal hyperproliferation, increased cleaved caspase-3 positive apoptotic keratinocytes were found in *Sharpin^cpdm^* skin with mitochondria defect [21]. 

The present study adds new lines of evidence of inflammation, proliferation, and apoptosis in the skin of *Sharpin^cpdm^* mice, a good tool to study NF*κ*B activation autoinflammatory diseases.

## 2. Materials and Methods

### 2.1. Mice

C57BL/KaLawRij-*Sharpin^cpdm^*/RijSunJ (JR no. 007599), and B6; 129S1-*Il1rap^tm1Roml^*/J (JR no. 003284) mice were maintained in a humidity, temperature, and light cycle (12 : 12) controlled vivarium under specific pathogen-free conditions (http://jaxmice.jax.org). *Sharpin^cpdm^* mice of both sexes are equally affected. As female mutant mice do not breed, they were used for all experiments unless indicated otherwise. Mice were housed in double-pen polycarbonate cages at a maximum capacity of four mice per pen. Mice were allowed free access to autoclaved food (NIH 31, 6% fat; LabDiet 5K52, Purina Mills, St. Louis, Mo) and acidified water (pH 2.8–3.2). All work was done with Institutional Animal Care and Use Committee approval.

### 2.2. Antibodies

Rabbit antimouse filaggrin (no. PRB-417P), keratin 5 (no. PRB-160P), keratin 14 (no. PRB-155P), keratin 1 (no. PRB-165P), keratin 10 (no. PRB-159P), and keratin 6 (no. PRB-169P) were from Covance (Richmond, Calif, USA). Monoclonal antialpha smooth muscle actin (no. A2547) from mouse was from Sigma (Saint Louis, Miss, USA). Rat antimouse Gr-1 (no. 550291), hamster antimouse CD3e (no. 550277) and rat antimouse CD45RB (no. 553097) were purchased from BD Biosciences (San Jose, Calif, USA). Alexa Fluor 488 goat antirat IgG (no. A11006), Alexa Fluor 546 goat antihamster IgG (no. A2111) and Alexa Fluor 546 donkey antigoat IgG (no. A11056) were purchased from Invitrogen Corporation (Carlsbad, Calif, USA). Horseradish peroxidase conjugated goat anti-rabbit IgG (no. 32460) and rabbit anti-goat IgG (no. 31402) were from Thermo Scientific (Rockford, Ill, USA).

### 2.3. Behavioral Tests

10-week-old mice were placed at set position in an open field and allowed to explore for 10 seconds. Distance traveled, holes explored, time in center, stretched attends, vertical explorations, number of fecal boli, and number of urinations were automatically recorded using the SMART system from San Diego Instruments (San Diego, Calif).

### 2.4. Immunohistochemistry

Dorsal skin was collected and fixed by immersion in Telly's solution. Fixed tissues were embedded routinely in paraffin and serially sectioned at 6 *μ*m. For routine histopathological examination, one section was deparaffinized, rehydrated, stained with hematoxylin and eosin, and then dehydrated in an alcohol series, cleared in Xylene, and mounted with Permount mounting media entellan (Fisher Scientific, Pittsburgh, Pa, USA). One section was stained with 0.5% acidified toluidine blue **(**in 0.5 M HCl; Merck, Germany) for 30 seconds. Twenty additional serial sections were placed on poly-L-lysine-coated slides for immunocytochemical staining. Paraffin sections were stained with special dilution for each antibody (keratin 5 @ 1 : 500, keratin 14 @ 1: 50, keratin 1 @ 1 : 2500, keratin 10 @ 1 : 1500, keratin 6 @ 1 : 2500, filaggrin @ 1 : 1500, alpha smooth muscle actin @ 1: 800, and caspase-3 @ 1 : 100).

### 2.5. Immunofluorescence

Dorsal skin was embedded in cryoprotectant (Tissue-Tek O.C.T., Sakura Finetek USA, Torrance, USA). Cryosections were prepared, fixed in ice-cold acetone, and blocked with 3% FBS (fetal bovine serum). Samples were then subjected to immunofluorescence analysis by successive 2-hour long incubations with primary (Gr-1 @ 1 : 100, CD3 @ 1 : 50, and CD45RB @ 1 : 100) and 1-hour long with secondary antibodies (1 : 2000) and finally counterstained with DAPI (1 : 10,000) for 5 minutes. Samples were examined using a laser scanning confocal microscope TCS-NT (Leica, Mannheim, Germany). 

### 2.6. Complete Blood Counts

Whole blood (275 *μ*L) was drawn from the retro-orbital sinus through EDTA-coated microhematocrit tubes directly into Eppendorf tubes containing 30 *μ*L 20% EDTA in PBS. The additional anticoagulant is critical in preventing microclot formation. Complete blood counts were determined immediately after obtaining the samples using an Advia 120 Multispecies whole blood analyzer (Bayer Corporation, Tarrytown, NY). Three *Sharpin^cpdm^* mutant and three +/+ control mice were used for each time point.

### 2.7. Transmission Electron Microscopy (TEM)

Strips of full-thickness skin samples (5 × 10 mm) were immersed immediately at the time of necropsy in cold fixative consisting of 2.5% glutaraldehyde and 2% paraformaldehyde in 0.1 M phosphate buffer (pH 7.2) and processed routinely. Pieces of the strips (1 × 2 mm) were transferred to fresh fixative overnight to 48 hours at 4°C. After a brief rinse in phosphate buffer, tissue specimens were postfixed in 2% phosphate-buffered osmium tetroxide, dehydrated through a graded series of EtOH, and embedded in Quetol 657 (EMS, Pa). Semithick sections (0.5 *μ*m) were stained with toluidine blue for light microscopy. Properly oriented blocks, with full thickness epidermis and dermis present, were used for ultrathin (100 nm) sections. Ultrathin sections were stained with uranyl acetate and lead citrate and examined in a JEM-1230 transmission electron microscope (Jeol USA Inc., Peabody, Mass).

### 2.8. Apoptosis Assays In Vivo and In Vitro

Apoptosis was evaluated on paraffin embedded sections of skin tissues by Tdt-mediated dUTP nick-end labeling (TUNEL) using In Situ Cell Death Detection Kit TMR red (Roche) per the manufacturer instructions. Rehydrated tissues were treated in 0.1 M citrate buffer, pH 6.0 with 350 W microwave irradiation for 5 min. Slides were then incubated with 50 *μ*L of TdT incubation buffer at 37 °C for 60 minutes inside a dark humid chamber. The reaction was terminated by immersing the slides in 2x standard saline citrate for 15 min. The tissue orientation was determined by final staining with 100 ng/mL DAPI solution. Apoptotic cells were visualized immediately with a confocal microscope, resulting in localized red fluorescence within the nucleus of the apoptotic cells.

Epidermal KCs were prepared from 6-week-old mice essentially per Lichti protocol [22]. For flow cytometric analysis, suspensions of KCs (1 × 10^6^) from 10 week old mice were fixed in 70% EtOH at 4°C. After fixation, cells were permeabilized with 0.01% Triton X-100 and centrifuged. The pellets were resuspended in PBS and treated with PE conjugated Annexin-V and propidium iodide. Cells were counted using flow cytometry (BD Biosience). Approximately 25,000 events were acquired for analysis using Cell Quest 3.3 Software (BD Biosience). A histogram plot of FITC fluorescence (*X* axis) versus counts (*Y* axis) was generated in logarithmic fluorescence intensity.

### 2.9. Measurement of Epidermal Thickness, Mitotic, and Apoptotic Keratinocytes

Epidermal thickness of 10 fields for each sample, one sample per mouse and 3 mice per group, was measured. Every field includes at least one full-length hair follicle as an internal standard for orientation. The epidermal thickness was measured from the basement membrane to the top of the stratum granulosum. Mitotic and apoptotic keratinocytes were counted from same fields of each sample.

### 2.10. Statistical Analysis

Data were expressed as means ± SE. Statistical comparisons between two groups were performed by the Student's *t*-test. Statistical significance was determined as *P* < .05.

## 3. Results

### 3.1. Excess Body Weight Loss and Reduced Behavior Mobility in Sharpin^cpdm^Mutant Mice

**Figure 1:**
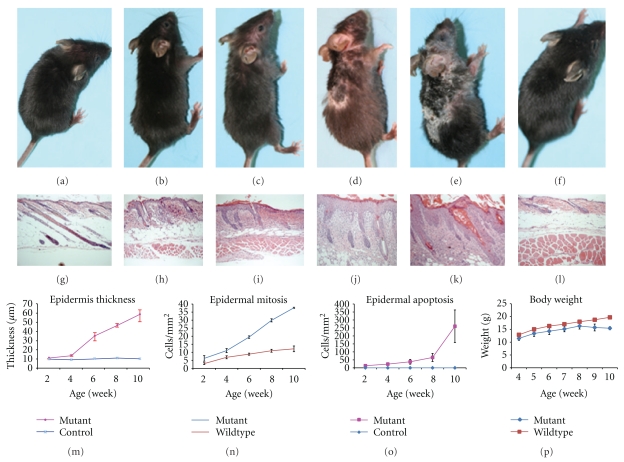
Chronic hyperproliferative dermatitis with loss of body weight. Longitudinal macroscopic observations at 5 time points (2-, 4-, 6-, 8-, and 10-week) found progressive development of dermatitis in *Sharpin^cpdm^* mutant mice (a)–(e), compared to the representative of wildtype mice at the age of 10 weeks (f). Hair loss and skin redness can be carefully found at forehead in 2-week-old mutant and gradually develop toward the posterior end of body. After the age of 6 weeks, pigmented skin islands formed containing broken hair shafts in skin and inflammatory pigmentation, covered with small, scurfy scales. Skin openings occur at the hairless areas after 6 weeks of age and gradually became deeper and wider until 10-week old. Histologically, increasing of epidermal thickness is consistent with the severity of epidermal hyperproliferation (g)–(m). Keratinocyte mitosis and apoptosis were quantitatively analyzed at each time point (m, n). Compared to wildtypes, the mutant mice present slower growth of body weight at the beginning of 4 weeks of age and lose body weight after 8 weeks of age (p).

**Figure 2:**
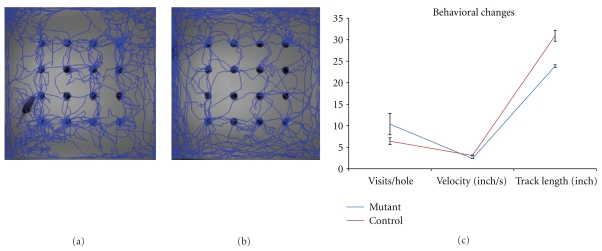
Exploratory test showed reduced behavioral activities in *Sharpin^cpdm^* mice. Mutant (a) and wild-type (b) mice were placed in open field with 16 holes on the board. Track distance, track speed, and hole visits were automatically recorded in limited time (c).

As shown in Figures 1(a)–1(e), hair loss and skin redness are usually recognized as first disease signs at the age of 2 weeks, and progressively become worse. Skin scales can be found and then accumulate at the erythematous areas. At the age of 8 weeks, skin appears to be crackly and ulcerated initially from hair loss area around the neck and foreleg and then spread to dorsal and ventral skin. The erythematous lesions become pigmented and scaling islands. Hairs are then broken from the skin surface, leaving the hair follicles in skin, which make separation of epidermis from dermis very difficult. Affected skin areas of 9-10 weeks mutant are then covered by psoriasis-like white scales and separated by deep ulcerations. Ophthalmoptosis and abscess under the neck skin can also be found in some mutants. Mutant females have congenital atresia of vagina (data not shown) and, therefore, are used for experiments. Mice showed poorer motor activity (2.38 inch/s) than controls (2.79 inch/s). Mutant mice mostly stay at the inner board and so track shorter length (23.86 inches), which may contribute to more hole visits (166) at the board central (Figures 2(a) and 2(c)), compared to 27.9 inches track and 102 visit holes of controls (Figures 2(b) and 2(c)). The longitudinal body weight of 3 pairs of mutant and healthy mice are obtained once a week from the age of 4 weeks to 10 weeks (Figure 1(m)). *Sharpin^cpdm^* mutant mice keep growing from 11.47 g at 4-week age to 16.3 g at the age of 8 weeks, and then lose weight to 15.44 g at 10-week age, when mice have to be euthanized regarding the disease severity. The wild-type mice are significantly bigger than the mutants from the age of 4 weeks (12.88 g) and keep growing to 15.03 g, 16.29 g, 17.02 g, 17.99 g, 18.74 g, and 19.71 g, respectively, at 5-, 6-, 7-, 8-, 9-, and 10-week age.

### 3.2. Severe Granulocyte Infiltration in the Skin of Sharpin^cpdm^ Mutant Mice

**Figure 3:**
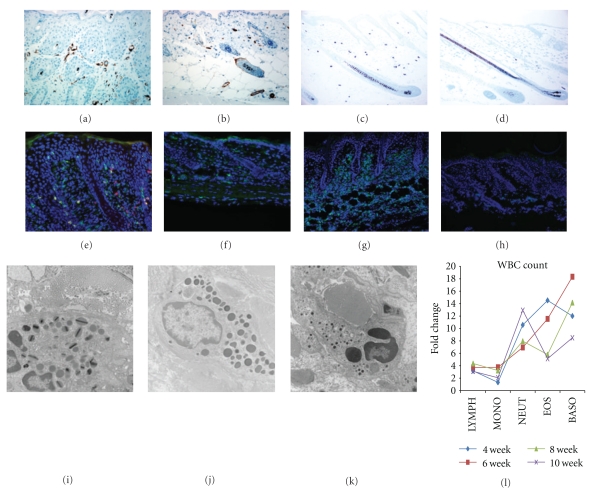
Granulocytic inflammation in the skin of *Sharpin^cpdm^* mice. (a, b): Alpha smooth muscle active was overexpressed in the skin blood vessel of mutant mice (a), compared to that of control (b). (c, d): Toluidine blue staining demonstrated increased infiltration of mast cells in the dermis of mutant mice (c). A few of mast cells present in the skin of healthy mice (d). (e, f): Increased T cells (Green, CD3e^+^) migrate into the skin lesion of mutant mice, among which some are helper T cells (double positive, CD3e^+^CD45RB^+^) (e). A few CD3e^+^T cells (Green), but no CD45RB^+^ T cells were detected in the normal control (f). (g)–(l): Gr-1^+^ granulocytes were clustered in the mutant skin (g), compared a few in normal skin for physiological demands (h). Transmission electron microscopy found most granulocytes are eosinophils (i), with moderate mast cells (j) and few neutrophils (k) although neutrophils had huge increase in peripheral blood (l).

Previous spectrum analysis of cytokines characterized a Th-2 inflammation in the skin of *Sharpin^cpdm^* mice [12]. As shown in Figure 3(e), double immunofluorescence staining using anti-CD3 (green) and anti-CD45 (red) has identified increasing of CD3^+^ cells, or CD45^+^ cells, or CD3^+^ CD45^+^ cells in the dermis of mutant mice, compared to a few CD3^+^ cells in the skin of control (Figure 3(f)). Cytokines and chemokines secreted by CD3^+^CD45^+^ helper T cells may contribute to the formation of Th-2 inflammation. The severity of inflammation is correlated with the over-expression of alpha smooth muscle actin in the mutant skin (Figures 3(a) and 3(b)). Toluidine blue staining confirmed huge increasing of mast cells in *Sharpin^cpdm^* dermis (Figures 3(c) and 3(d)). The number and morphology of red blood cells and platelets have no significant changes (data not shown). White blood cells in peripheral blood increase dramatically at every disease stage (Figure 3(l)). The granulocytes (eosinophils, basophils, and neutrophils) increase over 10-fold changes at average. Gr-1 positive inflammatory cells dramatically migrate into and form clusters in the mutant dermis (Figure 3(g)), while normally there are a few Gr-1^+^ cells in the skin of healthy mice (Figure 3(h)). Ultrastructure analysis have identified most of infiltrated granulocytes in mutant skin are eosinophils (Figure 3(i)), together with some mast cells (Figure 3(j)), and a few neutrophils (Figure 3(k)). Basophils are not found in the skin although it had 10 times of increasing in peripheral blood.

### 3.3. Increased Epidermal Thickness, Mitosis, Keratin, and Filaggrin Expression in Mutant Skin

**Figure 4:**
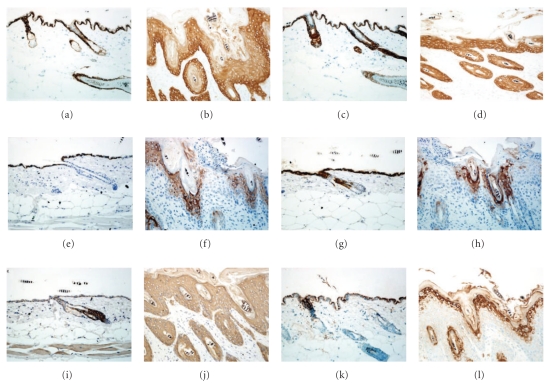
Expression changes of keratin and filaggrin in skin. (a, b): K5 expression in the basal layer of the epidermis of 10-week-old wild-type mice (a) but in the whole epidermis, preferentially in the basal layer, of 10-week-old *Sharpin^cpdm^* mice (b). (c, d): K14 expression in the basal layer of the epidermis of 10-week-old wild-type mice (c). K14 was uniformly expressed in the whole epidermis of 10-week-old *Sharpin^cpdm^* mice (d). (e, f): K1 expression locates in suprabasal layer of the epidermis of 10-week-old wild-type mice (e) but limited to the outer layers of the stratum spinosum of 10-week-old *Sharpin^cpdm^* mice (f). (g, h): K10 expression is located at the suprabasal layers of the epidermis and hair follicle of 10-week-old wild-type mice (g) but limited to the outer layers of the stratum spinosum of the epidermis and hair follicle of 10-week-old *Sharpin^cpdm^* mice (h). (i, j): K6 is only expressed in the hair follicle of 10-week-old wild-type mice (i) but widely expressed in both hair follicle and interfollicle epidermis of 10-week-old *Sharpin^cpdm^* mice (j). (k, l): Filaggin expression in the granular layers of the epidermis of 10-week-old normal and mutant mice.

As shown in Figure 1(n), the epidermal thickness remains constantly at 10 *μ*m after the age of 2 weeks in the skin of normal mice. It keeps increasing along with disease development from 10.9 *μ*m at 2-week stage to 59.17 *μ*m at 10-week age in *Sharpin^cpdm^* mutant mice. Correspondently, the mitotic basal cells increase slowly in the skin of healthy controls, respectively, of 2.93, 6.88, 8.83, 10.96, and 12.14 cells per mm^3^ at 2-, 4-, 6-, 8-, and 10-week age (Figure 1(o)). In comparison, huge increasing of mitotic basal cells is observed in the *Sharpin^cpdm^* mice, from 6.2 cells/mm^3^ at 2-week point to 37.71 cells/mm at 10-week point. In the skin lesion, the expression of keratinocyte differentiation markers in the skin of *Sharpin^cpdm^* mice are replicated here using antibodies provided by different vendors, compared to previous report. No quantitative differences in the changes in keratins and filaggrin are found between the 2-week-old and 10-week-old mice. K5 is primarily expressed in basal keratinocyte but also relatively lower in suprabasal layers of the epidermis of *Sharpin^cpdm^* mice (Figure 4(b)). K14 expression is uniformly distributed in all epidermal layers, without preferential expression in basal layer of the epidermis of *Sharpin^cpdm^* mice (Figure 4(d)). K1 is expressed in the suprabasal layers of the epidermis of normal mice (Figure 4(e)). However, K1 expression is only limited to the superficial cell layers of the stratum spinosum and inner shoot of hair follicle (Figure 4(f)). K10 expression is located in the suprabasal layers and outer shoot of hair follicle in the wildtype mice (Figure 4(g)). On contrast, K10 is only expressed in the superficial layers of the stratum spinosum and decreased expression in the hair follicle (Figure 4(h)). K6 is expressed in the hair follicle but not in the epidermis of skin of wild-type mice (Figure 4(i)). It is expressed in all layers of epidermis and hair follicles of 10-week-old *Sharpin^cpdm^* mice (Figure 4(j)). Filaggrin is detectable in granular layer of normal skin (Figure 4(k)). Its expression is increased in the granular layer, with variant expression in the spinous layer, of the *Sharpin^cpdm^* mice (Figure 4(l)).

### 3.4. Increased Single Cell Apoptosis of Keratinocyte in the Epidermis of Sharpin^cpdm^Mutant Mice

**Figure 5:**
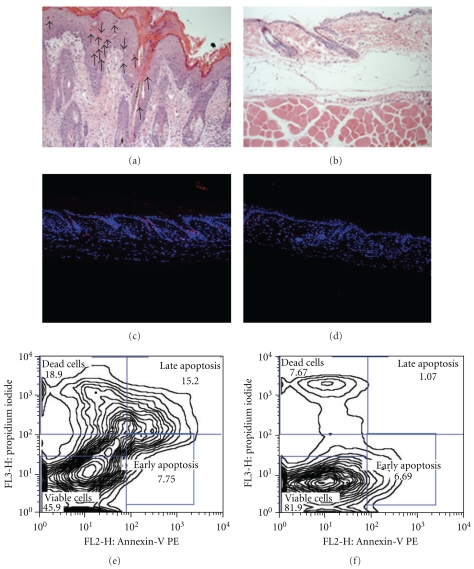
Increased keratinocyte apoptosis in the skin of *Sharpin^cpdm^* mice. (a, b): Numerous apoptosis bodies in the epidermis and hair follicles of 6-week-old *Sharpin^cpdm^* mice (a). No apparent apoptosis bodies were identified in the matched normal skin (b). (c, d): TUNEL immunofluorescence demonstrated that most apoptotic cells were located in the hair follicles, and some in the suprabasal epidermis, of 6-week-old *Sharpin^cpdm^* mice (c). No TUNEL positive cells were found in the epidermis of normal skin (d). TUNEL positive cells also present in the dermis of both mutant and normal skin. (e, f): Annexin-V/propidium iodide FACS analysis confirmed apoptosis in the epidermis of 6-week-old mutants. 15.2% and 18.9% of total epidermal cells of the mutant mice were, respectively, late apoptotic or actually dead cells (e), compared to 1.07% and 7.67% of the wild-type epidermal cells (f).

As counterpart of hyperproliferation and/or inflammation, increased apoptosis bodies in single epidermal cells were determined by H&E staining (arrowed in Figure 5(a)). Longitudinal quantitative analysis revealed epidermal keratinocytes death in *Sharpin^cpdm^* mutant skin increase geometrically from 13.5 cells/mm^3^ (2-week age) to 64.97 cells/mm^3^ (8-week age) and then steeply rise to 203.82 cells/mm^3^ at 10-week age (Figure 1(p)). TUNEL staining confirmed increasing of epidermal cell apoptosis, most of which were hair follicle keratinocytes (Figures 5(c) and 5(d)). However, TUNEL positive cells are also seen in the dermis of both mutant and wild-type mice, which is not detected by H&E staining, suggesting some false-positive cells by TUNEL staining. Epidermal cells from 6-week-old mutant and wild-type mice are isolated and sorted by Annexin-V and propidium iodide. Viable cells significantly decrease to 45.9% in mutant skin, compared to 81.9% viability in the matched control, indicating the increasing of apoptotic and/or dead cells in the *Sharpin*-defect skin (Figures 5(e) and 5(f)).

## 4. Discussion

Animal models are very important tools for studying the pathogenesis of human skin diseases [23, 24]. Complete phenotype analysis is first step to correlate and/or finally translate physiological or pathologic conditions. It has been reported that loss of function of SHARPIN in mice results in multiorgan inflammation, epidermal hyperproliferation, and mitochondria-mediated keratinocyte apoptosis [7, 11, 12, 21]. The present study have confirmed previous reports and added new lines of evidences to support these pathologic events. These novel data provide helpful clues for understanding and translation of this severe autoinflammatory disorder in mice.

 An expanding spectrum of acute and chronic inflammatory diseases is considered “autoinflammatory” diseases. This category of illness usually has a genetic defect, presents widespread inflammation, and lacks high-titer autoantibodies and antigen-specific T cells. Based upon underlying molecular mechanisms [25], six groups of autoinflammatory diseases were proposed as (1) IL-1*β* activation disorders, (2) NF*κ*B activation disorders, (3) protein folding disorders of the innate immune system, (4) complement disorders, (5) cytokine signaling disorders, and (6) macrophage activation disorders. The most recent study identified IL1 signaling was activated in the *Sharpin^cpdm^* skin, and blockade of IL1 signaling by knockout of IL1RAP can significantly alleviate the dermatitis severity [14]. Moreover, NF*κ*B signaling was activated primarily by stimuli including IL1 signaling, contributing to the development of skin problems in *Sharpin^cpdm^* mice, and NF*κ*B inhibitor, bortezomib, showed better therapy effect for dermatitis and mouse survival. The concentration of sera IgA, IgE, and IgG were significantly lower in *Sharpin^cpdm^* mice [26]. No autoantibodies and antigen-specific T cells were detected. Regarding the chronic systemic inflammation, *Sharpin^cpdm^* mutant mice may serve as an autoinflammatory mouse model of IL1 activation or NF*κ*B activation disorders. 

Chronic systemic inflammation has been found to at the root of many serious disorders, such as atopic dermatitis and psoriasis [27]. These “age-related” disorders are accompanied by a pathological increase of inflammatory cytokines. Type 2 inflammation was reported in the skin of *Sharpin^cpdm^* mice based on the increased expression of IL-4, IL-5, and IL-13 and no change of IL-10 and TNF [12]. Of interest is the increased infiltration of CD3e^+^CD45RB^+^ cells and the gradual increase of this subset in the *Sharpin^cpdm^* skin. This subset of cells may reflect accelerated differentiation of native CD45RA T cells under the influence of inflammation as suggested by the results on cytokine expression [28]. The high proportion of infiltrated granulocytes was eosinophils in *Sharpin^cpdm^* skin. Depending on the cytokines present in the microenvirohment, activated eosinophils can promote either a Th2 or Th1 immune response and amplify inflammation via autocrine fashion [29]. Considering apoptotic cells can selectively suppress the Th1 cytokines in peripheral blood mononuclear cells and shift the Th1/Th2 balance toward Th2, apoptotic keratinocytes in *Sharpin^cpdm^* skin may contribute to or ignite the Th2 inflammation as a local promoting factor [30]. A K14-cre conditional knockout mouse model will be the key tool to answer this question.

Chronic inflammation in animals and man acts like a tumor promoter, driving epithelial cell proliferation and epithelial carcinogenesis [31]. In *Sharpin^cpdm^* skin, mitotic keratinocytes increase constantly over time, along with similar pattern of epidermal thickness. The increased expression of K5 and K14 in the suprabasal layers indicates an increase of the proliferative compartment of the epidermis of mutant mice. Moreover, the presence of K6 expression in the interfollicular epidermis of *Sharpin^cpdm^* mice supports the epidermal hyperproliferation. The delayed expression of K1 and K10 in the outer layers of epidermis may reflect the accelerated suprabasal migration of keratinocytes, contributing to the excess accumulation of psoriasis-like white scales. Apoptosis balances proliferation to maintain epidermal thickness, contributes to stratum corneum formation, and may eliminate premalignant cells [32]. The disturbance between proliferation and apoptosis results in tumorigenesis and wound healing defect. Previous studies identified that loss of function of SHARPIN lead to caspase-dependent apoptosis via mitochondrial pathway [21]. In this present study, histological morphology quantitative analysis found consistent increasing apoptosis index in mutant mice as counterpart of hyperproliferation. Interestingly, TUNEL assay found that most of apoptotic keratinocytes located in the hair follicle, reflecting the pathogenesis of hair loss in the mutant mice. As hair loss is the first recognizable phenotype, keratinocyte apoptosis may serve as the primary drive of pathological events in mutant skin. It is possible that loss of function of SHARPIN results in mitochondria-mediated keratinocyte apoptosis, which contributes to the formation of Th2 inflammation and proliferation. Furthermore, the loss of body weight of the mutant may be consequence of the progressive loss of hairs (alopecia) and excess water loss from the disrupted skin barrier function.

## 5. Conclusion

The results of these studies provide novel clinical and molecular data of the *Sharpin^cpdm^* mutant mice and validate the pathological triad of inflammation, proliferation, and apoptosis in the skin of *Sharpin^cpdm^* mice. *Sharpin^cpdm^* mice may be a good tool to study NF*κ*B activation autoinflammatory diseases.
